# Fractal Analysis-Based Comparison of Mandibular Bone Remodeling in Clear Aligner and Fixed Orthodontic Therapies

**DOI:** 10.3390/jcm15145586

**Published:** 2026-07-16

**Authors:** Ömer Faruk Sari, Muhammed Hilmi Büyükçavuş, Elif Albayrak, Burak İncebeyaz

**Affiliations:** 1 Department of Orthodontics, Faculty of Dentistry, Ankara Medipol University, 06050 Ankara, Turkey; 2 Private Practice, Buyukcavus Dental Clinic, 03100 Afyonkarahisar, Turkey; mhbuyukcvs@gmail.com; 3 Department of Orthodontics, Faculty of Dentistry, Süleyman Demirel University, 32600 Isparta, Turkey; elfalbayrakk@gmail.com; 4 Department of Oral and Maxillofacial Radiology, Faculty of Dentistry, Ankara Medipol University, 06050 Ankara, Turkey; burakince06@hotmail.com

**Keywords:** fractal analysis, clear aligner therapy, fixed orthodontic treatment, mandibular trabecular bone, panoramic radiography

## Abstract

**Background:** To quantitatively compare treatment-related changes in mandibular trabecular bone between patients undergoing clear aligner therapy (CAT) and fixed orthodontic treatment (FOT) by means of fractal dimension analysis of panoramic radiographs. **Methods:** This retrospective investigation evaluated 76 patients who underwent orthodontic treatment with either clear aligner therapy (CAT; *n* = 34) or fixed orthodontic treatment (FOT; *n* = 42). Fractal dimension analysis was performed on panoramic radiographs obtained before treatment (T0) and after treatment completion (T1). Fractal dimension (FD) values were calculated for the mandibular molar, angulus, and condylar regions using ImageJ software based on the box-counting algorithm. Changes in FD were compared within and between groups using appropriate statistical tests, with statistical significance established at *p* < 0.05. **Results:** No statistically significant differences were observed between CAT and FOT groups regarding FD changes in the mandibular molar and angulus regions at T0 or T1 (*p* > 0.05). Within-group comparisons also revealed no significant FD changes in these regions following treatment in either group. In the condylar region, although FD values demonstrated a tendency toward change after treatment, intra-group differences were not statistically significant (*p* > 0.05). However, inter-group comparison revealed a statistically significant difference between CAT and FOT groups in condylar FD values (*p* ≤ 0.05), without evidence of destructive bone remodeling. **Conclusions:** CAT and FOT induce comparable adaptive responses in mandibular trabecular bone structure. FA findings suggest that both treatment modalities induce comparable adaptive remodeling patterns in mandibular trabecular bone without radiographic evidence of destructive bone changes, supporting the view that CAT could be considered a comparable alternative to FOT with respect to mandibular trabecular bone remodeling when appropriate patient selection and controlled force application are ensured.

## 1. Introduction

Orthodontic treatment aims to achieve esthetic and functional improvement by moving teeth into more favorable positions through mechanical and removable treatment modalities [1]. FOT has become one of the fundamental components of comprehensive orthodontic care, as it allows precise three-dimensional control of tooth movement [2]. Fixed appliances have been used as a conventional and effective orthodontic treatment modality for over a century. Over time, numerous studies have focused on enhancing their clinical efficiency, improving their biological and mechanical effects, and developing innovative approaches aimed at reducing overall treatment duration [3].

In recent years, increasing patient demand for more esthetic and comfortable orthodontic treatment options has led to a marked rise in the popularity of CAT [4]. The concept of CAT was first introduced by Kesling in 1946 as an approach for correcting irregular teeth [5]. Subsequently, the introduction of the Invisalign^®^ system by Align Technology, Inc. in 1998 enabled clear aligners to gain widespread clinical acceptance. In the early stages, these systems were primarily indicated for the treatment of mild crowding or spacing. However, advancements in material science and the development of computer-assisted tooth movement planning have substantially expanded the clinical applications of CAT [6]. Currently, a growing body of evidence indicates that CAT can provide successful treatment outcomes across a wide clinical spectrum, ranging from mild cases to more complex malocclusions [7,8].

FA has been widely used in dental research for various purposes due to its ability to detect changes in trabecular bone structure [9]. FD analysis, a mathematically based image-processing approach, enables the quantitative assessment of the effects of orthodontic treatment on alveolar bone [10]. In this method, images serve as analytical inputs, and the results are expressed as numerical data. Image processing is performed using computer-assisted analysis systems. The reliability of FA is highly dependent on standardized image quality, and it has been reported that high levels of image compression may adversely affect analytical accuracy [11].

Fractal analysis provides an indirect quantitative assessment of trabecular bone complexity by calculating the fractal dimension, which reflects alterations in bone architecture associated with remodeling processes. Unlike conventional radiographic interpretation, FA enables objective evaluation of subtle microstructural changes that may not be visually detectable. Because orthodontic tooth movement is accompanied by continuous bone resorption and apposition, FA has become an increasingly valuable method for monitoring biological responses to orthodontic treatment without exposing patients to additional radiation or requiring advanced imaging techniques [9,10].

Previous orthodontic studies have successfully used FA to evaluate alveolar bone remodeling after fixed appliance therapy, rapid maxillary expansion, functional appliances, and clear aligner treatment. These studies demonstrated that FA is capable of detecting adaptive changes in trabecular bone while remaining applicable to routinely obtained panoramic radiographs [11].

In recent years, FA has also been increasingly applied in orthodontic research. In addition to evaluating trabecular bone alterations, FD analysis has been widely used to assess bone mineral density using panoramic radiographs, with studies demonstrating a significant correlation between FD values and trabecular bone patterns [11]. Because panoramic radiographs are routinely obtained before and after orthodontic treatment, FA represents a non-invasive, practical, and accessible method for evaluating treatment-related changes in jawbone structure using existing radiographic records [9].

Because FA provides a quantitative, reproducible, and non-invasive assessment of trabecular bone microarchitecture using routinely acquired panoramic radiographs, it represents an appropriate method for evaluating treatment-induced bone remodeling during orthodontic therapy [9].

Previous studies have examined orthodontic bone remodeling using different imaging modalities and treatment protocols [9,10,11]. Nevertheless, no published study has directly compared the influence of CAT and FOT on mandibular trabecular bone using fractal dimension analysis. Therefore, the present study aimed to quantitatively assess treatment-related changes in mandibular trabecular bone by comparing panoramic radiographs obtained from patients treated with CAT and FOT. The results are expected to enhance current understanding of the biological response of mandibular bone to different orthodontic treatment modalities and may help inform future clinical treatment planning.

## 2. Materials and Methods

### 2.1. Study Design and Population

The present investigation was designed as a retrospective comparative study and conducted in accordance with the principles of the Declaration of Helsinki. Ethical approval was granted by the Clinical Research Ethics Committee of Ankara Medipol University (Approval No. 2026-25; 22 January 2026). The study aimed to quantitatively compare treatment-related changes in mandibular trabecular bone remodeling between CAT and FOT using fractal dimension analysis of panoramic radiographs.

This retrospective study included a total of 76 patients (43 females, 33 males) whose orthodontic treatment was completed at the Department of Orthodontics between January 2022 and December 2025, Faculty of Dentistry, Ankara Medipol University, and a private dental clinic. Of these patients, 34 were treated with CAT and 42 with FOT. Treatment modality was not randomly assigned because of the retrospective nature of the study. The choice between CAT and FOT was based on clinical indication, patient preference, esthetic expectations, compliance potential, and clinician recommendation. To reduce selection bias, only patients with mild-to-moderate malocclusions requiring comprehensive orthodontic treatment and without severe skeletal discrepancies were included. Patients with major skeletal disharmony, orthognathic surgery need, craniofacial anomalies, or systemic conditions affecting bone metabolism were excluded.

Both treatment groups were reviewed for baseline comparability regarding age, sex, treatment duration, malocclusion pattern, extraction status, and skeletal characteristics. Because of the retrospective nature of the study, detailed quantitative records regarding the degree of crowding and individual tooth movement patterns were not consistently available for all patients and therefore could not be included in the baseline comparison. Extraction and non-extraction cases were distributed between the groups as evenly as possible according to the available retrospective records. However, because treatment allocation was not randomized, possible selection bias cannot be completely eliminated and this has been acknowledged as a limitation.

Inclusion criteria were as follows: absence of congenital anomalies or a history of trauma, no previous orthodontic treatment, treatment carried out using either CAT or FOT protocols, and availability of high-resolution panoramic radiographs of sufficient diagnostic quality. Patients who did not meet any of these criteria were excluded from the study. To minimize potential confounding factors affecting mandibular bone remodeling, only patients with mild-to-moderate malocclusions requiring comprehensive orthodontic treatment were included. Cases requiring orthognathic surgery, severe skeletal discrepancies, or other conditions expected to substantially alter bone remodeling were excluded. Both treatment groups consisted of patients treated with similar orthodontic objectives using non-surgical comprehensive treatment protocols.

### 2.2. Sample Size

An a priori sample size calculation was performed using G*Power software (version 3.1.9.7; Heinrich Heine University, Düsseldorf, Germany). Based on an independent-samples *t*-test, assuming an effect size (Cohen’s d) of 0.80, an alpha error probability of 0.05, and a statistical power (1 − β) of 0.80, the minimum required sample size was calculated as 40 participants.

### 2.3. Radiographic Assessment and Treatment Protocol

Retrospective evaluation was performed using intraoral photographs together with digital panoramic radiographs retrieved from the institutional clinical database. Baseline panoramic radiographs (T0) were obtained immediately before the initiation of orthodontic treatment, whereas post-treatment panoramic radiographs (T1) were acquired immediately after completion of active orthodontic treatment. The interval between T0 and T1 corresponded to the duration of active orthodontic treatment, with a mean treatment time of 1.34 ± 0.51 years in the FOT group and 1.21 ± 0.59 years in the CAT group. These radiographs were selected for quantitative assessment of predefined mandibular regions. Image acquisition followed a uniform positioning protocol to ensure consistency among radiographs. During exposure, each patient was positioned with the Frankfort horizontal plane parallel to the floor while maintaining a stable bite on the positioning device. Imaging was carried out using a single panoramic radiography device (Planmeca ProMax^®^, Planmeca Oy, Helsinki, Finland) operating at 66 kVp, 10 mA, and an exposure time of 16 s. Digital images of uniform quality and resolution were preserved in the institutional archive and served as the basis for subsequent fractal analysis.

Patients were allocated into two groups according to the applied treatment protocol: CAT and FOT. In the CAT group, treatment was initiated following intraoral digital scanning, with aligner delivery and subsequent attachment placement. In the FOT group, treatment was initiated using 0.022-inch slot fixed appliances, followed sequentially by leveling and alignment, sagittal and transverse tooth movements, and finalized with a settling phase.

### 2.4. Fractal Analysis

Radiographic images were processed with ImageJ software (version 1.54p; National Institutes of Health, Bethesda, MD, USA), a freely available Java-based image analysis application. FD values were determined following the image-processing protocol proposed by White and Rudolph, in which the trabecular bone pattern is isolated before quantitative evaluation of its structural complexity [12].

Initially, regions of interest (ROIs) were identified, cropped, and converted to an 8-bit format to facilitate image processing. The mandibular molar, angulus, and condylar regions were selected because they represent anatomically and functionally distinct areas that are subjected to different biomechanical loading patterns during orthodontic treatment. These regions have also been commonly evaluated in previous fractal analysis studies, allowing direct comparison with the existing literature. To ensure measurement reproducibility, ROIs were selected according to predefined anatomical landmarks and standardized dimensions for all participants. The same ROI selection protocol was applied consistently to all panoramic radiographs by the same examiner throughout the study. To eliminate soft tissue effects and density variations and to enhance trabecular structures, image filtering procedures were applied. A duplicate of the original image was created, and a Gaussian blur filter with a sigma radius of 35 pixels was applied. The blurred image was then subtracted from the original image, and a grayscale value of 128 was added to prevent negative pixel values.

Subsequently, images were converted to binary format using thresholding to differentiate trabecular bone from marrow spaces. To reduce image noise and preserve trabecular continuity, erosion and dilation procedures were applied sequentially. The images were then inverted to allow optimal recognition of trabecular structures by the analysis software.

**Figure 1:**
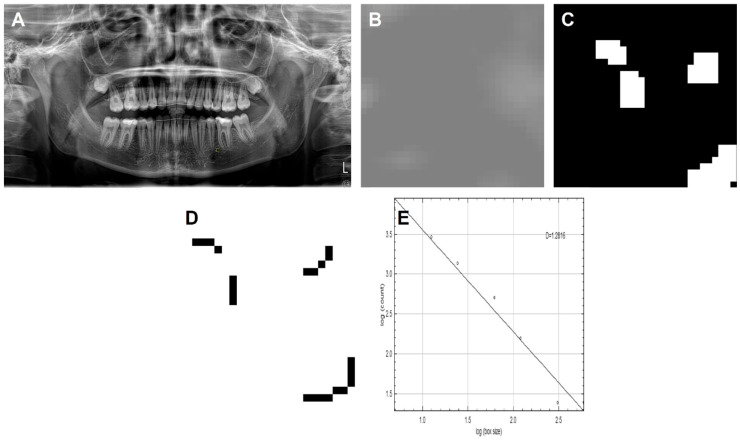
Fractal dimension analysis (FDA) procedure. (**A**) Original panoramic radiograph. (**B**) Gaussian-blurred image. (**C**) Binary image after thresholding. (**D**) Skeletonized image. (**E**) Fractal dimension calculation using the box-counting method.

In the final step, skeletonization was performed to reduce trabeculae to a single-pixel thickness, enabling evaluation of geometric complexity independent of trabecular thickness. FD values were calculated from the resulting skeletonized images using the box-counting method (Figure 1). To determine the consistency of the measurements, a subset of 20 panoramic radiographs was randomly chosen for repeated evaluation. All reassessments were carried out by the same investigator after a two-week interval using the same analysis protocol and measurement conditions. Measurement consistency was quantified by calculating intraclass correlation coefficients (ICC). The resulting ICC values (0.91–0.97) indicated a high level of intra-observer agreement and confirmed the reliability of the measurement protocol.

### 2.5. Statistical Analysis

The distribution of continuous variables was examined using the Shapiro–Wilk test. As the distribution of all variables satisfied the normality assumption (*p* > 0.05), subsequent analyses were conducted using parametric statistical methods. Categorical variables, including sex distribution and Class III malocclusion subtypes, were compared using the Pearson chi-square test. Independent-samples *t*-tests were used to compare demographic variables and between-group differences in baseline, post-treatment, and treatment-related changes (ΔFD), while paired-samples *t*-tests were applied to evaluate within-group changes between T0 and T1. A two-sided *p* value of <0.05 was considered indicative of statistical significance, while *p* values < 0.01 and <0.001 were interpreted as representing stronger levels of statistical evidence. Statistical analyses were carried out using IBM SPSS Statistics for Windows (Version 24.0; IBM Corp., Armonk, NY, USA). No formal correction for multiple comparisons was applied because the statistical analyses were predefined according to the study objectives and were considered exploratory. Therefore, the results should be interpreted in conjunction with the magnitude and consistency of the observed findings rather than statistical significance alone.

Because the primary objective of this retrospective exploratory study was hypothesis testing rather than estimation of treatment effects, effect sizes and confidence intervals were not routinely reported. Nevertheless, the observed findings were interpreted by considering not only statistical significance but also the magnitude and consistency of the treatment-related changes.

## 3. Results

The demographic profile and baseline skeletal characteristics of the participants are presented in Table 1. Group I (FOT) comprised 42 patients (23 females and 19 males), whereas Group II (CAT) included 34 patients (20 females and 14 males). The distribution of sex did not differ significantly between the groups (*p* > 0.05). Likewise, the mean chronological age was comparable, averaging 18.54 ± 1.96 years in the FOT group and 19.45 ± 1.77 years in the CAT group (*p* > 0.05). In contrast, treatment duration differed significantly between the treatment modalities, with a mean duration of 1.34 ± 0.51 years for FOT and 1.21 ± 0.59 years for CAT, indicating a shorter treatment period in the CAT group (*p* < 0.001).

Table 2 presents the FD values obtained at baseline (T0) and after treatment completion (T1), as well as treatment-related changes in the mandibular molar, angulus, and condylar regions in patients treated with FOT and CAT.

In the molar region, the FD value in Group I was 1.3868 ± 0.0372 at T0 and 1.3809 ± 0.0295 at T1, with no statistically significant change during treatment (*p* > 0.05). Similarly, in Group II, the molar region FD value was 1.3867 ± 0.032 at T0 and 1.3539 ± 0.0346 at T1, and the intra-group change was not statistically significant (*p* > 0.05).

In the angulus region, the FD value in Group I decreased from 1.368 ± 0.0308 at T0 to 1.3584 ± 0.0429 at T1, whereas in Group II, the FD values at T0 and T1 were 1.3593 ± 0.0368 and 1.3659 ± 0.0326, respectively. No statistically significant changes were observed in the angulus region in either group (*p* > 0.05).

In the condylar region, the FD value in Group I increased from 1.371 ± 0.0539 at T0 to 1.3783 ± 0.0419 at T1, with no statistically significant intra-group difference (*p* > 0.05). In Group II, the FD value increased from 1.3379 ± 0.0472 at T0 to 1.396 ± 0.0472 at T1; however, this increase did not reach statistical significance within the group (*p* > 0.05).

Overall comparisons of pre- and post-treatment FD values revealed no statistically significant differences within or between the groups in the mandibular molar, angulus, or condylar regions (*p* > 0.05).

Intergroup comparisons of treatment-related changes in fractal dimension (ΔFD) are presented in Table 3. The CAT and FOT groups showed comparable ΔFD values in the mandibular molar and angulus regions, with no statistically significant differences (*p* > 0.05). In contrast, a significant difference in ΔFD was observed in the condylar region between the two treatment modalities (*p* ≤ 0.05).

## 4. Discussion

The duration of orthodontic treatment varies depending on several factors, including the type of malocclusion, the need for tooth extraction, the application of surgical procedures, the presence of impacted teeth, systemic health conditions, and patient compliance with scheduled appointments [13]. In the present study, patients with any condition that could affect bone metabolism were excluded in order to minimize the influence of individual bone-related factors. Additionally, patients who did not attend follow-up appointments regularly were not included in the analysis.

Although CT-based imaging provides detailed visualization of bone architecture, its routine application in orthodontic practice remains limited because of the associated radiation dose [14]. CBCT has improved the three-dimensional assessment of craniofacial structures and is widely used when additional diagnostic information is required; nevertheless, it is not indicated for every orthodontic patient because radiation exposure should be justified according to clinical need [15]. In comparison, panoramic radiographs constitute a routine component of orthodontic records and have been demonstrated to provide reliable information on trabecular bone morphology for fractal dimension analysis [16]. Accordingly, panoramic radiographs were selected as the imaging modality for evaluating treatment-related changes in mandibular trabecular bone in the present study.

FD analysis enables the quantitative evaluation of changes in bone density using dental radiographs without the need for invasive procedures [17]. In contemporary dental research, FD analysis has been widely applied in various fields, including monitoring healing after endodontic treatment [18], assessing peri-implant bone structure [19], evaluating condylar changes in pediatric patients with bruxism [20], grading alveolar bone loss in individuals with periodontitis [21], analyzing the microstructural properties of restorative composite materials [22], and investigating the effects of orthodontic treatment on alveolar bone [23]. In the present study, FD analysis was applied to evaluate treatment-related changes in overall mandibular trabecular mineralization before and after orthodontic treatment with CAT and FOT. From this perspective, the present study extends the existing literature by directly comparing the effects of CAT and FOT on mandibular trabecular bone using fractal analysis under similar clinical conditions.

In this study, the effects of CAT and FOT protocols on trabecular bone structure in the mandibular molar, angulus, and condylar regions were compared using FA. The findings demonstrated that both treatment modalities produced generally similar bone remodeling patterns Overall, the findings indicate that both treatment modalities produced largely comparable trabecular bone responses in the evaluated mandibular regions. These findings are consistent limited FD changes in the mandibular condyle following FOT across different malocclusion groups [10]. Similarly, that FD changes observed after orthodontic treatment did not reflect destructive bone loss but rather corresponded to a physiological adaptation process [9].

CAT and FOT rely on different force delivery mechanisms to achieve tooth movement. While FOT applies more continuous and rigid forces, CAT systems deliver forces in a more intermittent and controlled manner. Although these distinct biomechanical approaches might theoretically be expected to result in different bone remodeling responses, the present study demonstrated similar FA outcomes in the mandibular molar, angulus, and condylar regions for both treatment groups. This finding is consistent with previous studies reporting limited and physiological FD changes in the mandibular condyle and alveolar bone following FOT [9,10]. Likewise, studies evaluating alveolar and mandibular trabecular structure using FD analysis after orthodontic treatment have consistently shown that controlled orthodontic forces do not induce destructive alterations in bone microarchitecture [24,25].

Recent investigations focusing on the effects of CAT on mandibular trabecular bone structure have also reported that clear aligner treatment does not result in significant bone loss in the condylar, gonial, or corpus regions [17,26]. These observations suggest that, despite differences in force application patterns, orthodontic forces applied within biological limits produce similar adaptive remodeling responses due to the intrinsic adaptive capacity of mandibular bone. Therefore, the present findings suggest that both CAT and FOT induce comparable adaptive trabecular bone responses without evidence of destructive remodeling within the limitations of the present study.

The mandibular molar region is directly exposed to orthodontic tooth movement and is one of the areas where alveolar remodeling is most pronounced. In the present study, the absence of significant differences in FD values in the molar region between CAT and FOT groups suggests that the applied orthodontic forces remained within physiological limits. This finding aligns with previous FA studies reporting that changes observed in alveolar trabecular structure after FOT are non-destructive in nature [11,24].

The angulus region, in contrast, is considered structurally more stable and is less directly influenced by orthodontic forces. The largely unchanged FD values observed in the angulus region in both treatment groups indicate a high resistance and adaptive capacity of trabecular bone in this area to mechanical loading. Previous studies reporting limited fractal changes in the angulus region following orthodontic or functional treatments further support these findings [10,27].

The condylar region, due to its close association with functional loading, occlusal changes, and masticatory patterns, is expected to exhibit the most dynamic adaptive responses during orthodontic treatment. Although a statistically significant inter-group difference was detected in the condylar region in the present study, this difference is not considered indicative of pathological remodeling. Similarly, Ertuğrul et al. reported that while treatment-related changes in condylar FD values may differ between orthodontic protocols, such changes remain measurable yet clinically limited throughout treatment [28]. In the present study, the condyle appeared to exhibit an adaptive response to orthodontic forces without evidence of structural deterioration or dramatic remodeling. Although a statistically significant inter-group difference was observed in the condylar region, the absolute magnitude of this difference was small and was not accompanied by evidence of destructive trabecular changes or clinically detectable adverse outcomes. Accordingly, this finding should be interpreted as a quantitative radiographic difference rather than evidence of clinically meaningful differences in mandibular bone remodeling between treatment modalities. Further prospective studies incorporating three-dimensional imaging and long-term follow-up are required to determine its clinical significance. Therefore, although the observed condylar difference reached statistical significance, its magnitude does not currently support a change in clinical decision-making between CAT and FOT.

Indeed, the literature consistently reports that FD changes observed in condylar trabecular structure following fixed orthodontic, clear aligner, or functional treatments are predominantly adaptive and reversible [10,11]. The limited differences observed in the condylar region in the CAT group may be related to adaptive changes in the condyle–fossa relationship associated with aligner thickness. Consequently, the observed condylar differences are likely reflections of treatment-related functional adaptation rather than adverse clinical outcomes. Nevertheless, not all studies have reported completely comparable findings. Differences in study design, patient age, treatment mechanics, imaging modality, region-of-interest selection, and fractal analysis protocols may contribute to variability among published results. Therefore, direct comparison between studies should be interpreted with caution.

Taken together, these findings indicate comparable adaptive remodeling responses following CAT and FOT. FA results demonstrated that neither treatment modality induced adverse changes in trabecular bone microarchitecture in the mandibular molar, angulus, or condylar regions. Clinically, these findings suggest that CAT could be considered a comparable treatment option with respect to mandibular trabecular bone remodeling when appropriate patient selection and controlled force application are ensured. Furthermore, FA enables practical and non-invasive monitoring of mandibular bone responses using routine panoramic radiographs. The excellent intra-observer agreement obtained in the present study indicates that the fractal analysis protocol was highly reproducible despite the operator-dependent nature of ROI selection.

The present study has several limitations that should be considered when interpreting the findings. First, although the sample size satisfied the a priori power analysis, the relatively limited number of participants may restrict the generalizability of the results. Second, fractal analysis was performed using two-dimensional panoramic radiographs, which are subject to image magnification, distortion, and superimposition of anatomical structures. Consequently, subtle trabecular changes may not have been fully captured. Furthermore, no CBCT validation was performed to confirm the radiographic findings obtained by fractal analysis. In addition, the study evaluated treatment-related changes only during active orthodontic treatment and did not include long-term post-treatment follow-up to assess the stability of mandibular trabecular bone remodeling.

Another limitation is the potential for selection bias due to the retrospective design and non-random allocation of patients to CAT or FOT. Although eligibility criteria were applied and baseline demographic characteristics were compared, treatment modality may have been influenced by patient preference, esthetic expectations, compliance, malocclusion characteristics, and clinician recommendation. Therefore, the findings should be interpreted with caution. Furthermore, detailed baseline orthodontic variables, including the degree of crowding, comprehensive malocclusion classification, and specific tooth movement patterns, were not consistently available because of the retrospective design. Therefore, the potential influence of these variables on trabecular bone remodeling could not be completely controlled.

Finally, the present findings were derived from a single retrospective cohort and have not been externally validated in independent populations. Therefore, future prospective multicenter studies are required to confirm the reproducibility and generalizability of these findings.

## 5. Conclusions

Within the limitations of this retrospective study, both clear aligner therapy and fixed orthodontic treatment demonstrated comparable adaptive changes in mandibular trabecular bone as assessed by fractal analysis. Although a statistically significant difference was observed in the condylar region, its clinical relevance remains uncertain. Therefore, the findings suggest that clear aligner therapy could be considered a comparable alternative to fixed orthodontic treatment with respect to mandibular trabecular bone remodeling. Nevertheless, prospective studies with larger sample sizes, three-dimensional imaging, and long-term follow-up are required to confirm these findings.

## Figures and Tables

**Table 1:** Demographic data of the groups.

	Gender Distribution ^†^ (Female/Male)	Chronological Age * (Years) Mean ± SD	Treatment/Follow-Up Duration * (Years) Mean ± SD
**Group I** **(FOT)** **Fixed Ortodontic Treatment**	23/19	18.54 ± 1.96	1.34 ± 0.51
**Group II** **(CAT)** **Clear Aligner Therapy**	20/14	19.45 ± 1.77	1.21 ± 0.59
***p***	NS	NS	NS

SD: Standard Deviation; ^†^: Pearson chi-square test; *: Independent *t* test; NS: Not significant.

**Table 2:** Changes in fractal analysis in different regions of the mandible and changes within the group.

	Molar Region x ± SD			Angulus Region x ± SD			Condyle Region x ± SD		
	T0	T1	*p′*	T0	T1	*p′*	T0	T1	*p′*
**Group I** **(FOT)** **Fixed Ortodontic Treatment**	1.3868 ± 0.0372	1.3809 ± 0.0295	NS	1.368 ± 0.0308	1.3584 ± 0.0429	NS	1.371 ± 0.0539	1.3783 ± 0.0419	NS
**Group II** **(CAT)** **Clear Aligner Therapy**	1.3867 ± 0.032	1.3539 ± 0.0346	NS	1.3593 ± 0.0368	1.3659 ± 0.0326	NS	1.3379 ± 0.0472	1.396 ± 0.0472	NS
***p*** **″**	NS	*	-	NS	NS	-	NS	NS	-

SD, Standard Deviation; NS, Not significant (*p* > 0.05); * *p* ≤ 0.05. *p′*, Paired *t*-test (intra-group changes); *p*″, Results of Independent *t*-test (Comparison of initial and post-treatment values across groups).

**Table 3:** Comparison of changes in fractal analysis in different regions of the mandible with treatment in groups.

	Molar Region x ± SD	Angulus Region x ± SD	Condyle Region x ± SD
**Group I** **(FOT)**	−0.0059 ± 0.0507	−0.0096 ± 0.0555	0.0073 ± 0.0617
**Group II** **(CAT)**	−0.0329 ± 0.0384	0.0066 ± 0.0563	0.0583 ± 0.0558
***p***	NS	NS	*

SD, Standard Deviation; NS, Not significant (*p* > 0.05); * *p* ≤ 0.05. *p*, Results of Independent *t*-test.

## Data Availability

The datasets generated and/or analyzed during the current study are available from the corresponding author upon reasonable request.

## Abbreviations

The following abbreviations are used in this manuscript:

CAT	Clear aligner therapy
FOT	Fixed orthodontic treatment
FA	Fractal analysis
FD	Fractal dimension
CBCT	Cone-beam computed tomography
